# Parallel phase 1 clinical trials in the US and in China: accelerating the test of avitinib in lung cancer as a novel inhibitor selectively targeting mutated EGFR and overcoming T790M-induced resistance

**DOI:** 10.1186/s40880-015-0029-3

**Published:** 2015-07-08

**Authors:** Xiao Xu

**Affiliations:** ACEA Biosciences Inc., San Diego, CA 92121 USA; ACEA Pharmaceutical Research, Hangzhou, Zhejiang 310030 P. R. China

## Abstract

Avitinib, a new generation inhibitor of epidermal growth factor receptor (EGFR), was approved for clinical trial in both China and the United States, and the phase 1 trials were initiated in both countries in parallel. In the preclinical studies, avitinib showed three novel features including (1) irreversibly binding *EGFR* by forming a covalent bound with Cys 797 in the ATP-binding pocket, (2) sparing wild-type *EGFR*, and (3) overcoming T790M-induced resistance. Avitinib is the first China-developed novel EGFR inhibitor that has entered in global clinical trials, and will provide a precision targeted therapy for non-small cell lung cancer patients.

The patients with non-small cell lung cancer (NSCL) containing somatic mutations of the epidermal growth factor receptor (*EGFR*) gene, such as the small deletions (747–750) and point mutations at codon 858 (L858R), are highly responsive to the currently marketed EGRF inhibitors [1–4]. These mutations on the *EGFR* gene mediate oncogenic effects by altering downstream signaling and antiapoptotic mechanisms, and such oncogenic effects can be inhibited by currently marketed EGFR inhibitors (e.g. gefitinib, erlotinib, and icotinib), which are together termed as the first-generation EGFR inhibitors. Despite the dramatic responses to such inhibitors, most patients ultimately have a relapse, and more than 50% of them developed a second point mutation resulting in threonine-to-methionine amino acid change at the position 790 of *EGFR* (T790M) [5], which is also termed as a gatekeeper mutation. The T790M mutation leads to the resistance against first-generation EGFR inhibitors. A newer irreversible EGFR inhibitor afatinib, so-called the second-generation EGFR inhibitor, has been recently approved by the Food and Drug Administration (FDA) in the United States (US). Although the inhibition of the T790M *EGFR* can be detected in the animal models and have been studied most frequently in the setting of acquired resistance to the first-generation EGFR inhibitors, the results from clinical studies did not clearly support that the treatment-resistant patients benefited from the second-generation EGFR inhibitor as a single drug treatment. In addition, because of the strong inhibition of wild-type *EGFR*, the second-generation EGFR inhibitor appears to have more adverse effects than first-generation EGFR inhibitors. Thus far, no standard therapeutic methods are available for the patients who relapsed after the treatment of first-generation EGFR inhibitors. New therapeutic intervention is urgently needed for the relapse.

In December 2009, a new generation EGFR inhibitor concept was reported by a group in Dana-Farber Cancer Institute [6]. The reported study aimed to explore a novel therapeutic method to overcome the T790M gatekeeper mutation-induced resistance after the treatment of first-generation EGFR inhibitors. In the study, an experimental compound, WZ4002, reported as a third-generation EGFR inhibitor, showed three novel features: (1) irreversibly binding *EGFR* by forming a covalent bound with Cys 797 in the ATP-binding pocket, (2) sparing wild-type *EGFR*, and (3) overcoming T790M-induced resistance. Shortly after the concept was published, we launched a program of developing a third-generation EGFR inhibitor as a novel therapeutic agent for overcoming the T790 M resistance, and the program was designed for both China and US FDA submission and global clinical trials at the very beginning. Therefore, in addition to meeting the high quality standard of science in the early discovery and development phase, fully understanding the regulatory guidance in China and US FDA and integrating the requirements from both regulatory agencies to the early development plan and strategy became the key to ensure the success of the preclinical studies and innovative new drug (IND) submission. Although both regulatory agencies require a very comprehensive preclinical IND data packages for innovative drugs, there are some differences. For the China FDA submission, the standards of Chemistry, Manufacturing, Control (CMC) package are almost close to the level of a final drug product, and the mechanisms of action and drug efficacy should be fully validated, whereas, for US FDA submission, the safety package including the full analysis of off-target effects of the compound and the clinical protocol should be well prepared to ensure the safety and ethic of the testing drug in human trials. In the preclinical studies, avitinib as an irreversible EGFR inhibitor showed the unique features of the third-generation EGFR inhibitor reported by the Dana-Farber group with strong potent inhibitory activities against *EGFR* bearing both active mutations and T790M mutations, and a better selectivity between wild-type *EGFR* and mutant *EGFR*. Avitinib has a great safety profile in rats and monkeys and can be orally administrated with good bioavailability and pharmacokinetic properties. The IND for avitinib was submitted to Center for Drug Evaluation (CDE), China FDA in August 2013 and approved for clinical trials in China in September 2014; in the US, the IND for avitinib was submitted to FDA in August 2014, and the clinical trial was granted in September 2014. The approval allowed avitinib clinical trials to be initiated in China and the US in parallel.

The clinical trials of avitinib were started in China and the US as the second-line therapy for NSCLC patients who have developed the resistance to first-generation EGFR inhibitors and acquired the gatekeeper mutation, T790M. In China, the clinical studies were started in Sun Yat-sen University Cancer Center, Guangdong People’s General Hospital, and Beijing Union Hospital (clinical pharmacology studies) for the dose escalation assessments on drug safety, pharmacokinetics, and efficacy. NSCLC patients with *EGFR* active mutations and having developed the resistance against first-generation EGFR inhibitors will be enrolled in the trials, and the T790M status will be further confirmed by genetic tests in biopsy tissue samples and blood samples. The multicenter trial will soon be initiated in China to expand the patient population at selected doses to further explore the safety and efficacy. In the US, the clinical studies were initiated in MD Anderson Cancer Center and other three university hospitals. The US phase I trial protocol is similar to the China trial protocol, and China clinical trial data are used as a reference for the US trials, such as the starting dose, the dose escalation design, dose schedules (once or twice a day), and possible adverse event control and monitoring schedules. To keep the consistence of the trial data, especially the experimental data, trials in both China and the US will use the same protocols, testing kits, and central labs. Thanks to the significant improvement in China clinical trials and participation in global clinical trials during last decade led by Chinese principal investigators and multinational pharmaceutic companies, we will be able to design the avitinib China trials as a part of the global trials soon being initiated in the US and other regions of the world. Therefore, avitinib development strategy will be able to be designed as an innovative drug not only for China but as an innovative global drug development program starting from China. At the time of this article’s submission, the clinical trials have followed the proposed trial timeline and patients have begun to benefit from the treatment of avitinib.

Conducting clinical trials in China and the US for an innovative drug developed in China is challenging and costly, whereas such trials have been considered an absolutely necessary step towards globalization of the China-innovated drugs. The successful IND approval and initiation of clinical trials for avitinib in both China and the US may offer a breakthrough therapeutic opportunity to address the urgent needs for NSCLC patients not only in China but also outside China. In addition, the ongoing global clinical development of avitinib will further enrich our knowledge and experiences of how to improve the global development process of domestically developed anticancer drugs.
